# Fatal Hæmorrhage from Tooth Extraction

**Published:** 1887-09

**Authors:** 


### Fatal Hæmorrhage from Tooth Extraction. [Cocaine and Opium Poisoning.]

C. Edmunds Kells, jr. D.D.S., New Orleans, reports (*N. O. Med. and Surg. Journal*, July, 1887,) a case of fatal hæmorrhage from gums some eight hours after extraction of fourteen teeth. It does not appear that during all this time as much as ten or twelve ounces of blood were lost, though no estimate is given by the *Reporter*. Seven and a half minims of a four per cent. solution of cocaine were injected in gums about 11.30 a. m. Seven minutes later ten minims more were injected. This was followed at once by "local applications of a perhaps 50 per cent. solution of the same drug to the gums." Extraction was begun twenty minutes later. At once, patient had spasmodic contractions of muscles of body and face, which were so violent as to prevent application of forceps, except with difficulty, and at length ten teeth and four roots were extracted. His restlessness continued; injected 15 minims camphor water, which seemed to quiet him; amyl nitrite was also inhaled. Hæmorrhage ceased; a toddy was given and patient discharged about 1 p. m. About 3.30 p. m. the dentist was sent for to stop the hæmorrhage [which had begun again from the sockets]. Patient was very excitedly talking, walking up and down the room, spitting blood in *very large* quantities. Injected 15 minims camphor solution in gums. As he would not sit down nor stop sucking out pledgets put in the sockets, plugging failed. At 4 o'clock his physician tried plugging, but failed for same reason as above. Iron and alum mouth wash failed, until it was made ice cold. Attempt to inject morphine, gr. $\frac{1}{8}$ to $\frac{3}{16}$ths was only partially successful, because of the patient's constant movements, but at last became quiet and laid down. Restlessness returned in a short time, and a quarter grain morphine was injected subcutaneously, "after which he quieted, immediately becoming very pale, although his pulse felt 'full and strong.'" At 7 p. m. he was quietly lying on sofa. A few minutes later his physician was recalled and found him breathing once a minute. Once under electric current, breathing returned to 13 per minute, but it then fell back until it finally ceased at 1 a. m.

